# Career Perspectives of Michael N. Sawka

**DOI:** 10.1186/2046-7648-1-10

**Published:** 2012-11-07

**Authors:** Michael N Sawka

**Affiliations:** 1 Thermal & Mountain Medicine Division, US Army Research Institute of Environmental Medicine, Kansas Street, Natick, MA 01760-5007, USA

**Keywords:** Thermoregulation, Physiology, Performance, Research career, Hydration, Environmental medicine

## Abstract

This invited autobiography reviews the career of Michael N. Sawka. **Influences**: Dr. Sawka soon will retire after a 40-year research career and was influenced by great professors, mentors and colleagues. **Career Path**: After working at the Dayton Veterans Administration Medical Center and Wright State University, Dr. Sawka’s remaining 32 years were at the US Army Research Institute of Environmental Medicine. **Research Story**: His primary research thrusts included: 1) physiology of upper body exercise; 2) blood volume and its impact on thermoregulation and performance; 3) hydration and its impact on thermoregulation and performance 4) heat stress physiology - adaptations / maladaptations and performance. **Summary:** His career highlights were the personal interactions, intellectual excitement and satisfaction of producing knowledge that will be “tested by time”.

## Influences

**Figure 1:**
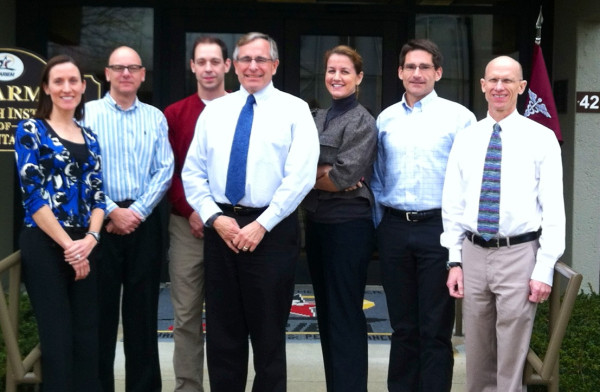
The USARIEM “heat stress performance and hydration team” of Brett Ely, John Castellani, Sam Cheuvront, Mike Sawka, Nisha Charkoudian, Bob Kenefick and Scott Montain (left to right) in March 2012.

It is difficult to imagine that I will be soon retiring. The rapidity with which ~40 years of research has passed is testimony to the incredible enjoyment I received from my career. I was fortunate to be influenced by great professors (Drs. Neil Anderson and Herb Weber at East Stroudsburg University; Drs. Jerry Critz and Ron Knowlton at Southern Illinois University), mentors (Drs. Carl Gisolfi, John Greenleaf, Ethan Nadel, Kent Pandolf), colleagues (Drs. Ralph Francesconi, Roger Glaser, Rich Gonzalez, Dan Miles, Bruce Wenger, Andy Young) and many bright young scientists (e.g., Figure 1). Most importantly I was blessed with a wonderful wife who always supported me and quietly tolerated the long hours, a father who demanded a strong work ethic, and a mother who encouraged me to be inquisitive and enjoy thinking.

An important inspiration for my future research came during the summer of 1974 when I studied with Dr. Bruno Balke (Aspen Institute). Dr. Balke pioneered exercise stress testing and was a prominent environmental medicine scientist. He introduced me to the fascination of studying physiological integration by imposing an environmental (thermal and hypoxic) stress upon a human engaged in physical exertion. This was the genesis of my research theme of “tweaking” the human body with multiple stressors to help reveal physiological mechanisms of compensation, adaptation and maladaptation.

## Career path

I never planned a career path, it just evolved from a series of events. I was raised in Northern New Jersey and Eastern Pennsylvania and had a vague idea of being a coach, biology teacher or Physical Therapist. My family were Coca-Cola bottlers so as a teenager I delivered soda to East Stroudsburg University (ESU), which was in our territory, and became friendly with many coaches, faculty and administrators. Therefore, ESU was my natural college choice and I immediately became interested in physiology through the exciting lectures of Dr. Neil Anderson. I then met Dr. Herb Weber who mentored me and employed me in the Cardiac Rehabilitation Program. As a result, from my second undergraduate year on, I was immersed in some aspect of exercise physiology. When graduating (BS degree), I was accepted into a Physical Therapy program and planned to go that route. Dr. Weber encouraged me to stay at ESU for my MS degree, as I already completed >50% of the graduate credits, and to later apply to Ph.D. programs. Dr. Weber and Dr. Ron Knowlton from Southern Illinois University (SIU) were friends, and Ron Knowlton taught a summer course at ESU. As a result, I developed a deep respect for Dr. Knowlton and chose SIU for my Ph.D. studies. SIU was a wonderful experience with great mentoring from Ron Knowlton and the recently arrived Jerry Critz. My dissertation was on dehydration, thermoregulation and cardiovascular control during prolonged exercise [1,2]. It is interesting that I would return to many of these research topics later in my career.

At the 1977 American Physiological Society meetings, I met Dr. Roger Glaser who offered me a post-doctoral fellowship at the Dayton Veterans Administration Medical Center to study the health and exercise capabilities of wheelchair dependent patients. Later I moved, across town, to a faculty position at Wright State University School of Medicine and this provided an opportunity to broaden my research scope (with collaborators Roger Glaser, Dan Miles and Jerry Petrofsky) to include skeletal muscle metabolism and upper body exercise. The combination of isometric with dynamic exercise and use of a small skeletal muscle mass posed many interesting physiology problems and the research findings resulted in my first review paper [3].

In 1980 I moved to the US Army Research Institute of Environmental Medicine (USARIEM, Natick, MA) to become a full-time research scientist. USARIEM provided an opportunity to study environmental physiology and move back to the “East Coast”. USARIEM had great facilities and tradition (preceding scientists such as Drs. Dave Bass, Clark Blatteis, Ellsworth Buskirk, G. Edger Folk, Mel Fregley, L. Howard Hartley, A.R. Lind) that were extremely motivating. Most importantly, USARIEM provided the opportunity to conduct environmental research that had direct bearing on the health and safety of courageous young men and women who risk their lives for our freedom. This fundamental reality facilitated a culture of methodical and solid science upon which to base military doctrine. At USARIEM, Drs. Kent Pandolf and Ralph Francesconi provided me great mentoring and friendship. In addition, we were later enriched when Drs. Richard Gonzalez and Bruce Wenger moved to USARIEM from the J.B. Pierce Foundation Laboratory at Yale University. Those were exciting times and culminated in publishing our graduate textbook on Environmental Medicine [4], the writing of which was a scientifically broadening experience. I continued at USARIEM assuming a variety of senior positions, but my love was always the science and the interaction with my collaborators.

I was asked to comment on who I influenced. I am unsure who I have influenced, but am very proud of my post-doctoral fellows (Steve Muza, Keith Prusaczyk, Scott J. Montain, John Castellani, Bill Latzka, Sam Cheuvront, Robert Carter III and David DeGroot) and the numerous students who worked in our laboratories and later become accomplished physiologists (e.g., Rob Boushel. Nisha Charkoudian, Catherine Gabaree, Darrel Neufer, Dae Taek Lee).

## Research story

It would be inappropriate to write my research story without fully acknowledging the wonderful contributions of USARIEM, of which I have been only a small part [5,6]. My research primarily focused on three broad topics: (1) blood (plasma and erythrocyte) volume and its impact on thermoregulation and performance [7], (2) hydration impact on thermoregulation and performance [8], (3) and heat stress physiology - adaptations / maladaptations and performance [9]. In addition, I collaborated with Drs. John Castellani, Steve Muza, Andy Young and others on adaptations and fatigue mechanisms associated with cold and high-altitude stressors [10,11].

**Figure 2:**
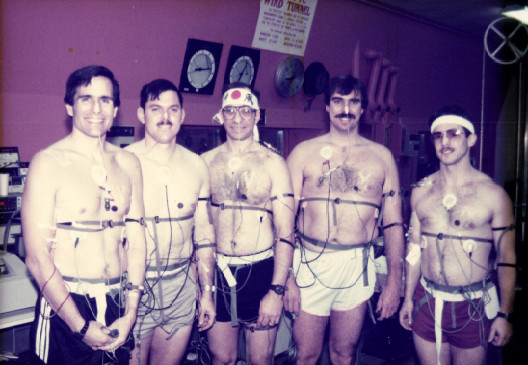
Volunteers Mike Sawka, Rich Weringo, Steve Muza, Andy Young and Bill Latzka (left to right) for the erythrocyte volume expansion and dehydration study conducted in December 1987.

For me the most fascinating studies were those which independently manipulated erythrocyte volume, plasma volume and hydration to determine their independent effects on thermoregulation and performance [7,8]. Critical collaborators included Drs. Robert Valeri and Richard Dennis at the Naval Blood Research Laboratory (then at Boston University Medical School). My favorite study examined the role of erythrocyte volume expansion on thermoregulation, in which we found the greater the perturbation (dehydration) the greater the benefits during exercise-heat stress [12]. Figure 2 shows myself and fellow subjects of that study - in those days you could participate in your own experiments. Unfortunately, I discontinued my research on erythrocyte volume expansion because political sensitivity dissuaded the funding of subsequent grant proposals.

If I were a young investigator, there would be many research questions which I would like to follow up. Larry Sonna determined that prior viral infection might predispose individuals for exertional heat stroke [13]. Dr. Lisa Leon is continuing research on this “multiple-hit” hypothesis that suggests such an exposure might neutralize the protective molecular adaptations from heat acclimation [9]. Research on the potential health impact of dehydration has not progressed because there is no single valid measure of hydration status [14]. Dr. Sam Cheuvront (see Figure 1) is developing (with industrial partners) technologies for a valid non-invasive hydration status measure. We believe that exercise performance in the heat is impaired because of high skin blood flow requirements [15] and hypovolemia [16] via the cardiovascular system rather than a “critical core temperature” as is a popular belief in the sports medicine literature [17]. Drs. Bob Kenefick and Nisha Charkoudian (see Figure 1) are continuing research to determine if blood pressure control (challenged by vasodilation and hypovolemia) provides the critical signals for impairing exercise-heat performance. There are many unanswered questions regarding heat acclimation and associated molecular adaptations [18] and their impact on exercise performance in temperate conditions [19]. The questions regarding molecular adaptation to heat acclimation are being addressed by USARIEM investigator Dr. James McClung collaborating with Dr. Jeff Hasday at the University of Maryland Medical School; while heat acclimation and performance in temperate environments questions will hopefully be followed-up by Ms. Brett Ely (see Figure 1) who is starting her Ph.D. with Dr. Chris Minson (advisor to Dr. Santiago Lorenzo who conducted the most complete study on this topic) at the University of Oregon.

## Summary

I have thoroughly enjoyed my career, have no regrets, and would not change any aspect of it. My advice to students is to seek a career path that you really enjoy and that matches your personality. Science takes incredibly long hours and “thick skin”, but the personal interactions, intellectual excitement and satisfaction of producing knowledge that will be “tested by time” is incredibly fun and rewarding.

## Abbreviations

ESU: is East Stroudsburg University; SIU: is Southern Illinois University; USARIEM: is United States Army Research Institute of Environmental Medicine.

## Competing interests

I have no competing interests.

## Disclaimer

The opinions or assertions contained herein are the private views of the authors and should not be construed as official or reflecting the views of the Army or the Department of Defense.

## References

[B1] Sawka MN Knowlton RG Critz JB Thermal and circulatory responses to repeated bouts of prolonged running Med Sci Sports 1979 11 177 180 491877

[B2] Sawka MN Knowlton RG Glaser RM Body temperature, respiration, and acid–base equilibrium during prolonged running Med Sci Sports Ex 1980 12 370 374 7453517

[B3] Sawka MN *Physiology of upper body exercise* Exercise and Sport Sciences Reviews. Volume 14 1986 New York: Macmillan 175 211 3525185

[B4] Pandolf KB Sawka MN Gonzalez RR Human Performance Physiology and Environmental Medicine at Terrestrial Extremes 1986 Indianapolis, IN: Cooper Publishing Group

[B5] Francesconi R Byrom RT Mager M United States Army research Institute of Environmental Medicine: first quarter century Physiologist 1986 29 58 62 3534911

[B6] Pandolf KB Francesconi RP Sawka MN Cymerman A Hoyt RW Young AJ Zambraski EJ US army research institute of environmental medicine: warfighter research focusing on the past 25 years Advances in Physiological Education 2011 35 353 360 10.1152/advan.00049.2011 22139770

[B7] Sawka MN Young AJ *Acute polycythemia and human performance during exercise and exposure to extreme environments* Exercise and Sport Sciences Reviews. Volume 17 1989 Baltimore: Williams & Wilkins 265 293 2676549

[B8] Sawka MN Coyle EF Holloszy JO Influence of body water and blood volume on thermoregulation and exercise - heat performance Exercise and Sport Sciences Reviews. Volume 27 1999 Baltimore, MD: Williams and Wilkins 167 218 10791017

[B9] Sawka MN Leon LR Montain SJ Sonna LA Integrated physiological mechanisms of exercise performance, adaptation and maladaptation to heat stress Comprehensive Physiology 2011 1 1883 1928 23733692 10.1002/cphy.c100082

[B10] Castellani JW Sawka MN Degroot DW Young AJ Cold thermoregulatory responses following exertional fatigue Front Biosci 2010 2 854 865 10.2741/s106 20515829

[B11] Young AJ Sawka MN Moravec J, Takeda N, Singai PK Blood volume changes during altitude acclimatization: implications for aerobic performance Adaptation Biology and Medicine. Volume 3 2002 New Delhi: Narosa Publishing House 191 201

[B12] Sawka MN Gonzalez RR Young AJ Muza SR Pandolf KB Latzka WA Dennis RC Valeri CR Polycythemia and hydration: effects on thermoregulation and blood volume during exercise-heat stress Am J Physiol 1988 24 R456 R463 Regulatory, Integrative and Comparative Physiology) 3414840 10.1152/ajpregu.1988.255.3.R456

[B13] Sonna LA Wenger CB Flinn S Sheldon HK Sawka MN Lilly CM Exertional heat injury and gene expression changes: a DNA microarray analysis study J Appl Physiol 2004 96 1943 1953 10.1152/japplphysiol.00886.2003 14978005

[B14] Cheuvront SN Ely BR Kenefick RW Sawka MN Biological variation and diagnostic accuracy of dehydration assessment markers Am J Clin Nutr 2010 92 565 573 10.3945/ajcn.2010.29490 20631205

[B15] Ely B Cheuvront SN Kenefick RW Sawka MN Aerobic performance is degraded, despite mild hyperthermia, in hot environments Medicine and Science in Sports and Exercise 2009 42 135 141 20010120 10.1249/MSS.0b013e3181adb9fb

[B16] Kenefick RW Cheuvront SN Palombo LJ Ely BR Sawka MN Skin temperature modifies impact of hypohydration on aerobic performance J Appl Physiol 2010 109 79 86 10.1152/japplphysiol.00135.2010 20378704

[B17] Sawka MN Cheuvront SN Kenefick RW High skin temperature and hypohydration impairs aerobic performance Exp Physiol 2012 97 327 332 22143882 10.1113/expphysiol.2011.061002

[B18] McClung JP Hasday JD He J Montain SJ Cheuvront SN Sawka MN Singh IS Exercise-heat acclimation in humans alters baseline levels and *ex vivo* heat-inducibility of HSP72 and HSP90 in peripheral blood mononuclear cells Am J Physiol 2008 294 R185 R191 10.1152/ajpregu.00532.2007 17977914

[B19] Lorenzo S Halliwill JR Sawka MN Minson CT Heat acclimation improves aerobic performance J Appl Physiol 2010 109 1140 1147 10.1152/japplphysiol.00495.2010 20724560 PMC2963322

